# Vasopressin in vasoplegic shock in surgical patients: systematic review and meta-analysis

**DOI:** 10.1590/acb387523

**Published:** 2023-12-01

**Authors:** Taís Felix Szeles, Juliano Pinheiro de Almeida, José Arnaldo Shiomi da Cruz, Everson Luiz Almeida Artifon

**Affiliations:** 1 Universidade de São Paulo – Medical School – Anesthesiology Department – São Paulo (SP) – Brazil.

**Keywords:** Atrial Fibrillation, Norepinephrine, Mortality, Hospitalization, Vasoplegia, Vasopressins

## Abstract

**Purpose:**

Vasoplegia, or vasoplegic shock, is a syndrome whose main characteristic is reducing blood pressure in the presence of a standard or high cardiac output. For the treatment, vasopressors are recommended, and the most used is norepinephrine. However, new drugs have been evaluated, and conflicting results exist in the literature.

**Methods:**

This is a systematic review of the literature with meta-analysis, written according to the recommendations of the PRISMA report. The SCOPUS, PubMed, and ScienceDirect databases were used to select the scientific articles included in the study. Searches were conducted in December 2022 using the terms “vasopressin,” “norepinephrine,” “vasoplegic shock,” “postoperative,” and “surgery.” Meta-analysis was performed using Review Manager (RevMan) 5.4. The endpoint associated with the study was efficiency in treating vasoplegic shock and reduced risk of death.

**Results:**

In total, 2,090 articles were retrieved; after applying the inclusion and exclusion criteria, ten studies were selected to compose the present review. We found no significant difference when assessing the outcome mortality comparing vasopressin versus norepinephrine (odds ratio = 1.60; confidence interval 0.47–5.50), nor when comparing studies on vasopressin versus placebo. When we analyzed the length of hospital stay compared to the use of vasopressin and norepinephrine, we identified a shorter length of hospital stay in cases that used vasopressin; however, the meta-analysis did not demonstrate statistical significance.

**Conclusions:**

Considering the outcomes included in our study, it is worth noting that most studies showed that using vasopressin was safe and can be considered in managing postoperative vasoplegic shock.

## Introduction

Vasoplegic syndrome (VS) is characterized by systemic arterial hypotension associated with standard or increased cardiac output and reduced systemic vascular resistance. Increased need for fluids or vasopressors represents the more severe systemic inflammatory response syndrome spectrum. The occurrence rate ranges from 5 to 25% in postoperative patients without known risk factors. Still, in those with a known predisposition to the syndrome, the prevalence can range from 30 to 50% of cases^1^.

In this regard, vasoplegia is present in advanced shock states, including septic, cardiogenic, hemorrhagic, and anaphylactic shock^2^. It presents a complex pathophysiology that involves several mechanisms in vascular smooth muscle cells, such as desensitization of receptors coupled to the G protein (adrenoceptors, vasopressin receptor, type 1 angiotensin receptor), alteration of second messenger pathways, acute corticosteroid insufficiency and increased production of nitric oxide^3^^–^ 5.

The most common therapeutic approaches for vasoplegia predict the need for changes in treatment, with the application of personalized multimodal treatment with the indication of various vasopressors. Although norepinephrine is considered a first-line therapy for the treatment of vasoplegia, recent guidelines from the Surviving Sepsis Campaign think that the best therapeutic management of vascular hyporesponsiveness to vasopressors could be a combination of multiple vasopressors, including norepinephrine, and early prescription of vasopressin^6^. The inclusion of this new approach may be justified by limiting adrenoceptor desensitization and sympathetic hyperactivation due to their subsequent deleterious impacts on hemodynamics and inflammation^7^.

In postoperative cardiac surgery, the recent Vasopressin versus Norepinephrine in Patients with Vasoplegic Shock after Cardiac Surgery, a randomized, controlled, double-blind study, compared vasopressin to norepinephrine in vasoplegic shock after cardiac surgery^8^. This study used vasopressin as the first treatment option for shock, in direct comparison with norepinephrine. This study showed that the group randomized to use vasopressin showed reduction in a combined endpoint of mortality and severe complications, a decrease in the intensive care unit (ICU) length of stay, and a lower incidence of supraventricular arrhythmias^1^.

Although some studies show benefits in using vasopressin, the Surviving Sepsis Campaign 2016 recommendations on catecholamine therapy clear norepinephrine as the vasopressor of first choice. Vasopressin, as an additionally administered drug, is classified as more tentative. Evidence for the benefit of vasopressin remains low, with reduced incidence of newly diagnosed atrial fibrillation and reduced mortality rate. Still, the administration remains safe^9^. Therefore, we performed a systematic review followed by metanalysis to evaluate the efficacy of using vasopressin in treating postoperatively vasoplegic syndrome, especially considering the mortality outcome.

## Methods

The current meta-analysis was performed according to the recommendations of the PRISMA statement^10^. It was registered in PROSPERO under the number CRD 42020208622.

### Search strategy

We searched for four databases for studies, i.e., PubMed, Embase, Web of Science, and Google Scholar. In addition, we also looked for any undergoing trials on clinicaltrials.gov. The keywords searched were “vasopressin,” “norepinephrine,” “Vasoplegic syndrome,” and “surgery.” There was no restriction on our search by language. The bibliographic details of all the included studies were searched manually for any additional citations. In case of duplication of publication, the analysis with the entire data set was included.

### Eligibility criteria

Inclusion criteria were randomized controlled trials, and observational studies, published from 2012 to 2022, whose clinical outcome was using vasopressin compared to norepinephrine or placebo in treating vasoplegic shock. The main characteristics of the studies analyzed in the meta-analysis included patients aged 18 years old or older undergoing high-risk cardiac surgery and postoperative patients of non-cardiac surgeries. We considered as high-risk non-cardiac surgery all abdominal, thoracic, and orthopedic surgical procedures with an expected duration greater than 90 minutes and in which the patient presented at least one of the following high-risk criteria: age over 65 years old, coronary artery disease, severe left ventricular dysfunction (defined as ejection fraction less than 30% by echocardiography), moderate or severe heart valve disease, heart failure, chronic obstructive pulmonary disease, chest radiography showing chronic lung disease, chronic renal failure (creatinine ≥ 1.5 mg/dL), and diabetes mellitus.

We excluded publications that did not correspond to the research topic; publications that did not present a complete text; repeated publications; publications that addressed the issue tangentially to the objective of this study; publications that addressed case reports or case series; publications that were not finalized (“preprint”); literature reviews, and those that did not meet the previously mentioned inclusion criteria.

### Assessment of bias risk

The quality of scientific evidence in the studies covered was classified as high, moderate, low, or very low according to the risk of bias in the body of evidence, clarity of comparisons, precision, and consistency in treatment effects. High-quality evidence was assigned to well-designed randomized controlled trials with consistent results. The quality of evidence was considered moderate if 1 of the four quality of evidence criteria was not met, and low if two or more were not met. A low rate of evidence was assigned to studies with inconclusive results. Cochrane Collaboration’s tool was used to check for the quality of studies included in the meta-analysis^10^.

### Data synthesis and statistical analysis

For data analysis, a database was built in Microsoft Excel spreadsheet that was later exported to the statistical program Minitab 18 (version 18, Minitab, LLC, State College, Pennsylvania, United States of America) (Minitab) and also to OriginPro 9 (DPR Group, Inc., Northampton, Massachusetts, United States of America) (Moberly, Bernards, Waynant, 2018).

For the statistical analysis, we extracted the data from the selected original articles obtaining the values of total N, mean, standard deviation, 95% confidence interval (95%CI), and percentage (frequency) for all predictors. One-way analysis of variance (ANOVA) test was applied, adopting the α level less than 0.05 with a statistically significant difference for the 95%CI. The value of R-sq (I2) was analyzed to determine the analyses’ imprecision or heterogeneity. The codes of low association = < 25%, medium association 25% < X < 50%, and high association = > 50% were adopted. For dichotomous outcomes, the estimated effect of the intervention was expressed as risk ratio (RR) and odds ratio (OR), along with 95%CI. Relevant data were extracted from the studies, and the Mantel-Haenszel statistical method was used for data synthesis in the Rev Man 5.4 program.

## Results

### Literature search

**Figure 1:**
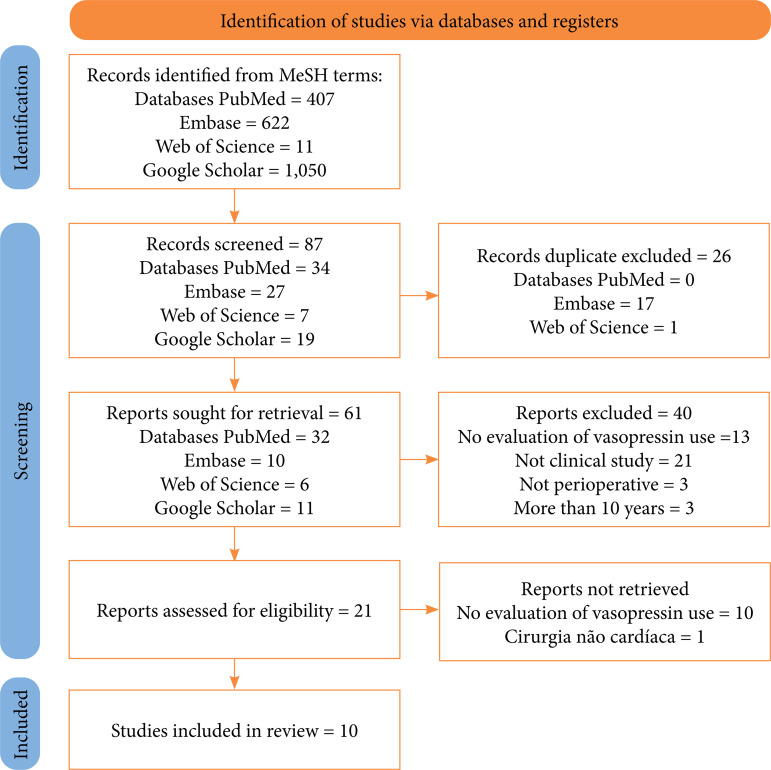
PRISMA diagram with the results found in the research.

Source: PRISMA^11^.

A total of 2,090 articles were obtained in the initial search, 87 studies involving vasopressin and VS. Twenty-six duplicate articles were removed, leaving 61 for screening. After screening titles and abstracts, ten full-text articles remained for review (Fig. 1 and Table 1).

**Table 1:** Characteristics of the studies selected for the metanalysis.

Authorship (year)	Type of study	Patients number	Groups	Inclusion criteria	Exclusion criteria	Start of intervention	Primary outcome	Secondary outcome	Conclusion
Hajjar et al. (2017)^3^	Randomized trial	300	Vasopressine (149)	Cardiac surgery	Aortic surgery, transplant	Up to 48 hours CPB output	Mortality	Infection, septic shock	First randomized study vasopressin as first choice
			Norepinephrine (151)	> 18 years old	Preoperative vasopressor use		severe complications	Arrhythmia-atrial fibrillation	Reduced incidence of serious complications
					Severe hyponatremia		In 30 days	Length of stay and intensive unit care	Shorter length of stay and intensive unit care
					Re-infarction, mesenteric ischemia			Renal Failure	Reduced incidence of atrial fibrillation
									Vasopressin may be preferable to norepinephrine
Okamoto et al. (2015)^12^	Randomized trial	92	Vasopressine (47)	Cardiac Surgery > 20 years old	No intervention	Intraoperative	Elevation of cardiac enzymes	Atrial fibrillation	Stabilized hemodynamic parameters
			Placebo (45)		Serious adverse event during the surgery	anesthetic induction		Vasoplegia during surgery	Did not elevate cardiac enzymes
						Prophylatic		Renal Failure	Did not change surgical outcome
								length of stay and intensive unit care	
								Mortality	
Jahangirifard et al. (2017)^13^	Randomized trial	80	Vasopressine (40)	CRV elective surgery	Ejection fraction > 50 and < 35%	30 minutes before	Intensive unit care stay	Use of dopamine	Reduced required dose of dopamine
			Placebo (40)		Renal Failure or hepatic dysfunction	From the CPB exit	Hazard ratio and blood pressure measurement	Vent duration mechanics	Decreased heart rate
					Epilepsy		Urinary output		Did not change length of stay
					emergency surgery		Arrhythmia		
Dargah et al. (2018)^14^	Randomized trial	120	Vasopressine (60)	CRV surgery	Without RF, Creatinine > 1.5	Intraoperative	Kidney function		Norepinephrine had better result in renal function
			Norepinephrine (60)	with CPB	Diabetes				Sodium, Potassium, Urea and Creatinine levels showed no difference
				> 30 years old	Arterial hypertension				
Verma et al. (2022)^15^	Randomized trial	60	Vasopressine (30)	CRV surgery	Ejection fraction < 30%	After anesthetic induction	Hemodynamic monitoring	Adverse effects	Prophylactic low-dose infusion is safe
			Placebo (30)	without CPB	Emergency surgery			Cardiac enzyme elevation	Cardio and nephroprotective
				Age between 30 and 70 years old	Other surgeries			Kidney function	Better hemodynamic profile and less blood loss
					Diabetes mellitus, failure liver and renal				Decreased need for ionotropic drug
Elgebaly and Sabry (2012)^16^	Randomized trial	20	Vasopressine (10)	CRV surgery	ejection fraction < 30%	10 minutes before	Hemodynamic monitoring	Length of stay and intensive unit care	Prophylactic low-dose infusion
			Placebo (10)	ejection fraction between 35 and 50%	Presence of prior shock	From the CPB exit			Reduce dose of catecholamines
					Dysfunction hepatic, adrenal and renal	Holds up to 60 minutes			Prevents incidence of vasoplegic shock
					Carotic stenosis	from the CPB exit			Decreased CPB side effects
Eissa (2014)^17^	Randomized trial	60	ACEi + Placebo (20)	CRV elective surgery	Valvopathy or congestive heart failure	20 minutes before start	Hemodynamic monitoring	Duration Ventilation mechanics	Use of vasopressin is more beneficial than norepinephrine
			ACEi + Norepinephrine (20)	With CPB	Dysfunction left ventricle moderate or severe	of the CPB		Length of stay and intensive care unit	Low-dose vasopressin preventive vasoplegic shock
			ACEi + Vasopressine (20)	ACEi use (lisinopril)	Prior shock			Need for transfusion	Decreased time on Ventilation mechanics and intensive care unit stay
				Normal LV function or mild disfuction	Pulmonary hypertension			Use of vasoactive drugs	
Porhomayon et al. (2015)^18^	Retrospective control case	483	Vasopressine (280)	CRV surgery	Other cardiac surgery	Undefined	Insufficiency acute renal (AKIN-1)	Mortality	99% male patients (Veterans Hospital)
			Without Vasopressina (203)		Off-pump surgery creatinine > 1.5 or RF stage 3	Perioperative		Stroke/sepsis E-infarction	Vasopressin has been associated with greater kidney damage
Bomberg et al. (2016)^19^	Observational cohort	78	Vasopressine + Norepinephrine (11)	Mesenteric ischemia		Intensive unit care	Mortality	E-infarction	Data before angiography, after 24 and 48 hours of treatment
			Norepinephrine (67)	Non-occlusive				Length of stay and intensive unit care	Benefit in the use of vasopressin associated with Nora
				after heart surgery					Improvement of small intestine perfusion
Cheng et al. (2018)^20^	Retrospective cohort	338	Vasopressine (169)	Cardiac surgery		Intensive unit care admission	Mortality within 30 days	Infection, septic shock	Atrial fibrillation and arrhythmias were higher in the vasopressin group
			Norepinephrine (169)	> 18 years old	Congenic cardiopatics		Vent. Mechanics > 48 hours	Atrial fibrillation	No improvement of results
				Ejection fraction < 35%	Acute mesenteric ischemia		Cardiac re-operation	Arrhythmias	
				diameter left ventricular diameter > 60 mm	Pregnancy, cancer		Stroke and acute kidney injury		
				Post operative vasoplegic shock	Use extracorporeal membrane oxygenation				

CRV: cardiac revascularization; RF: renal failure; CPB: cardiopulmonary bypass; ACEi: angiotensin converting enzyme inhibitor; LV: left ventricle. Source: Elaborated by the authors.

### Qualitative synthesis

Seven clinical trials and three observational studies were included. In a double-blind, randomized clinical trial published by Okamoto et al.^12^, the aim was to investigate the relationship between intraoperative vasopressin and the presence of postoperative cardiac enzymes. One hundred patients who underwent cardiac surgery were included. These patients were randomized into two groups. In the first group, patients used vasopressin (1.8 U/h), and in the second group, they used a placebo (saline 1.8 mL/h). The primary endpoint was an assessment of CK-MB and troponin T levels at the end of surgery, and 6 and 12 hours afterward. The authors found no significant differences in the levels of the markers evaluated at any time during the study.

Hajjar et al.^3^ conducted a double-blind, randomized clinical trial in which patients with vasoplegic shock after cardiac surgery were included. These patients were randomized into two groups. Group 1 (n = 149) was infused with vasopressin, and group 2 (n = 151) with norepinephrine. The primary endpoint was mortality or severe complications, and 32% of patients in the vasopressin group died, while in the norepinephrine group the rate was 49% (hazard ratio–HR = 0.55; 95%CI 0.38–0.80; p = 0.0014).

Jahangirifard et al.^13^ also conducted a double-blind, randomized clinical trial to evaluate the prophylactic effect of low-dose vasopressin in patients undergoing cardiac revascularization. They included 80 patients randomly divided into two groups. In the first group (n = 40), patients received 0.03 IU/min 30 minutes before the end of cardiac revascularization. In the second group (n = 40), patients received saline solution. The authors found that vasopressin administration decreased the required dopamine dose (p = 0.031) after cardiac surgery.

Dargah et al.^14^ conducted a randomized clinical trial to compare the incidence of renal failure in patients undergoing coronary artery revascularization who used vasopressin or norepinephrine. One hundred and twenty randomized patients were included in the two groups. The authors demonstrated a statistically significant difference only considering creatinine clearance, which was higher in the group that received norepinephrine compared to the group that received vasopressin (p < 0.05).

Verma et al.^15^ performed a double-blind, randomized clinical trial to evaluate the prophylactic effect of vasopressin administration in patients undergoing coronary artery bypass. In this study, 60 patients were randomly divided into two groups, one group received vasopressin (0.03 IU/min via infusion pump), and the other group received a saline infusion pump. Heart rate and cardiac output were lower in the group receiving vasopressin, and mean arterial pressure and systemic vascular resistance were higher in this group. Central venous pressure was stable with no significant difference. The authors concluded that low-dose vasopressin infusion is safe for postoperative vasodilatory shock.

Elgebaly and Sabry^16^, was a double-blind, randomized study to assess whether low doses of vasopressin administered in patients undergoing coronary graft revascularization with mild to moderate systolic dysfunction can produce improvement in cardiac function. Twenty patients were randomly divided into two groups; the patients received 0.03 IU/min of vasopressin in the second saline solution in equal volume for 60 minutes. The authors demonstrated that infusion of low doses of vasopressin for patients with systolic dysfunction benefits the postoperative hemodynamic profile, reduces the doses of catecholamines required, and improves left ventricular systolic function.

The final one, performed by Eissa^17^, included clinical trial, in a randomized double-blind study, in patients undergoing elective cardiac revascularization with cardiopulmonary bypass (CPB), who used lisinopril, an angiotensin-converting enzyme inhibitor, pre-operatively. Sixty patients were included, divided into three groups: 20 patients did not receive any medication, 20 patients received norepinephrine 0.03–0.05 mcg/kg/min, and 20 patients received vasopressin 0.03 IU/min. The intervention began 20 minutes before the start of prophylactic CPB. The primary outcome was hemodynamic monitoring. The result showed that low-dose vasopressin is beneficial with an improvement in the hemodynamic profile and less blood loss.

Considering observational studies, Porhomayon et al.^18^ evaluated the effect of vasopressin on postoperative acute renal failure. A retrospective cohort study was conducted, in which 483 patients undergoing coronary revascularization were included. Patients were grouped according to preoperative vasopressin use, and the development of acute renal failure was the primary outcome. According to the results, the authors showed that the incidence of renal failure in the total sample was 14.5%. Vasopressin was administered in 280 patients, and the prevalence of renal failure was 20% in this group and 6.1% in the patients who did not receive it (p < 0.0001). Moreover, the use of vasopressin was an independent factor in predicting the occurrence of renal failure (OR = 3.6; 95%CI 1.22–10.62; p = 0.02). However, this association needed to be recovered in propensity score matching analysis (p = 0.073).

Bomberg et al.^19^ also conducted an observational cohort study in which 78 patients undergoing cardiac revascularization who had non-occlusive mesenteric ischemia as a complication were included. Among these patients, in 11 the blood pressure was not maintained with the use of norepinephrine alone. Therefore, vasopressin was administered. Two days after treatment, the patients who used vasopressin improved bowel perfusion compared to those who did not (p = 0.002). In addition, all patients in the vasopressin group survived, and 17 patients in the group that did not use vasopressin died in the hospital.

Cheng et al.^20^ conducted a retrospective observational cohort study in which 1,156 patients who had vasoplegic shock after cardiac surgery and who used vasopressin or norepinephrine were included. The primary endpoint was mortality rate, mechanical ventilation for more than 48 hours, stroke, acute renal failure, and extracorporeal membrane oxygenation. Propensity matching analysis was performed, and, after it, 338 patients were selected (169 used vasopressin and 169 used norepinephrine). No significant differences were found in any of the primary outcomes assessed. However, when evaluating atrial fibrillation and ventricular arrhythmias, the rates were statistically significantly higher in the patients who used vasopressin (p = 0.038 and p = 0.014, respectively).

### Quantitative synthesis

**Figure 2:**
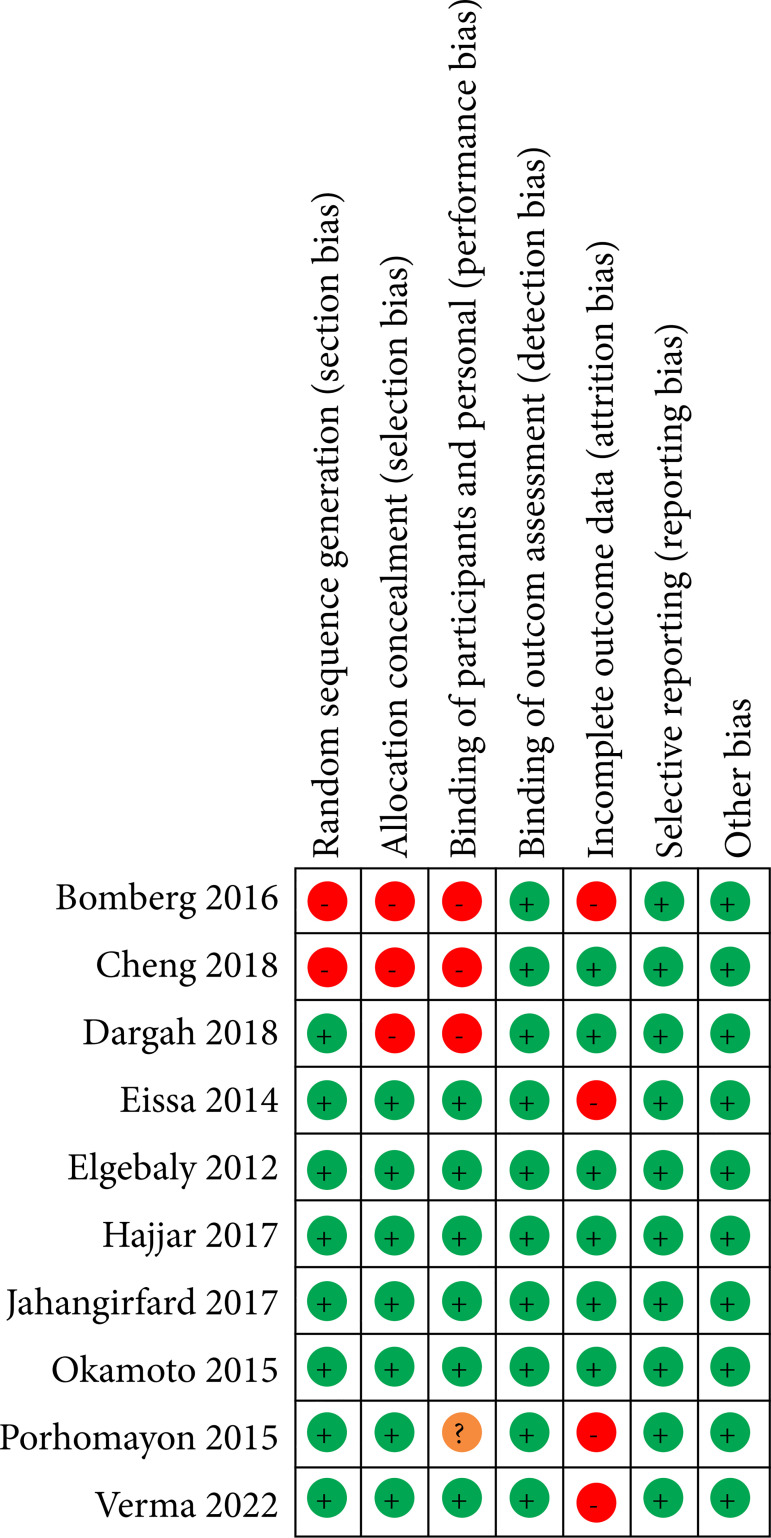
Risk of bias analysis of all studies included in the meta-analyses.

Source: Elaborated by the authors.

**Figure 3:**
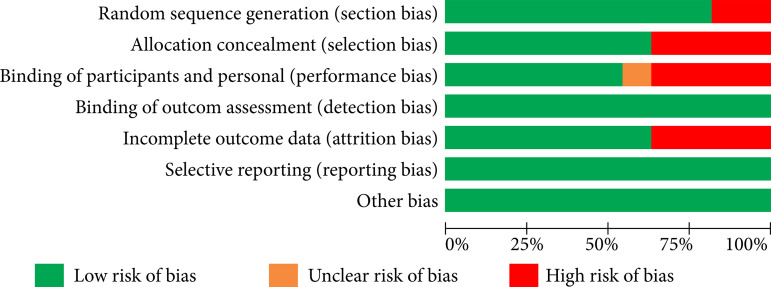
Risk of bias item presented as percentages across all included studies.

Source: Elaborated by the authors.

The bias risk analysis of all studies included in the metanalyses is shown in Fig. 2. We used the Cochrane RoB 2.0 tool to perform the risk analysis of the studies, and the results are shown in Fig. 3. Regarding selection bias, we noticed 20% of high risk for randomization and 40% for allocation. For performance bias, there was 40% high risk and for attrition bias 40% high risk. The quality of the evidence was assessed using GRADE, and this result is demonstrated in Tables 2 and 3.

**Table 2:** Quality of the evidence was assessed using GRADE for mortality.

**Vasopressin compared to norepinephrine for mortality in patients with vasoplegic shock after cardiac surgery**					
**Patient or population:** Mortality in patients with vasoplegic shock after cardiac surgery **Intervention:** vasopressin **Comparison:** norepinephrine					
Outcomes	N of participants (studies) Follow-up	Certainty of the evidence (GRADE)	Relative effect (95%CI)*	Anticipated absolute effects	
				Risk with norepinephrine	Risk difference with vasopressin
Vasopressin vs. noreprinefrine	638 (2 RCTs)	⊕⊕⊕ High	OR 1.60 (0.47 to 5.50)	84 per 1,000	44 more per 1,000 (43 fewer to 252 more)
Vasopressin vs. placebo	655 (3 RCTs)	⊕⊕⊕◯ Moderate	OR 0.72 (0.14 to 3.61)	10 per 1,000	3 fewer per 1,000 (9 fewer to 26 more)
**GRADE Working Group grades of evidence** **High certainty:** we are very confident that the true effect lies close to that of the estimate of the effect. **Moderate certainty:** we are moderately confident in the effect estimate: the true effect is likely to be close to the estimate of the effect, but there is a possibility that it is substantially different. **Low certainty:** our confidence in the effect estimate is limited: the true effect may be substantially different from the estimate of the effect. **Very low certainty:** we have very little confidence in the effect estimate: the true effect is likely to be substantially different from the estimate of effect.					

* The risk in the intervention group (and its 95% confidence interval) is based on the assumed risk in the comparison group and the relative effect of the intervention (and its 95%CI); 95%CI: 95% confidence interval; OR: odds ratio; RCT: randomized clinical trial. Source: Elaborated by the authors.

**Table 3:** Quality of the evidence was assessed using GRADE for length of stay.

Vasopressin compared to placebo for length of hospital stay					
Patient or population: length of hospital stay Intervention: vasopressin Comparison: placebo					
Outcomes	N of participants (studies) Follow-up	Certainty of the evidence (GRADE)	Relative effect (95%CI)*	Anticipated absolute effects	
				Risk with placebo	Risk difference with vasopressin
Vasopressin vs. norepinephrine	377 (4 RCTs)	High	RR -0.04 (-0.34 to 0.25)	0 per 1,000	0 fewer per 1,000 (0 fewer to 0 fewer)
Vassopressin vs. placebo	192 (3 RCTs)	Moderate	OR 0.81 (0.42 to 1.57)	611 per 1.000	51 fewer per 1,000 (214 fewer to 101 more)
**GRADE Working Group grades of evidence** **High certainty:** we are very confident that the true effect lies close to that of the estimate of the effect. **Moderate certainty:** we are moderately confident in the effect estimate: the true effect is likely to be close to the estimate of the effect, but there is a possibility that it is substantially different. **Low certainty:** our confidence in the effect estimate is limited: the true effect may be substantially different from the estimate of the effect. **Very low certainty:** we have very little confidence in the effect estimate: the true effect is likely to be substantially different from the estimate of effect.					

* The risk in the intervention group (and its 95% confidence interval) is based on the assumed risk in the comparison group and the relative effect of the intervention (and its 95%CI); 95%CI: 95% confidence interval; OR: odds ratio; RR: risk ratio; RCT: randomized clinical trial. Source: Elaborated by the authors.

### Mortality

**Figure 4:**
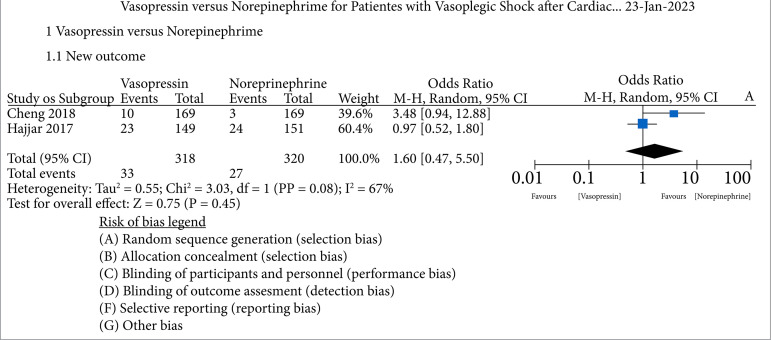
Mortality rate as endpoint comparing patients receiving vasopressin and norepinephrine.

In our analysis, we included studies that evaluated the mortality rate as an outcome comparing patients who received vasopressin and norepinephrine. As we can see in the forest plot (Fig. 4), two studies were included in this analysis, and the mortality rate was higher in the vasopressin group, although without statistical significance in the meta-analysis (OR = 1.60; 95%CI 0.47–5.50, I2 = 67%; z = 0.75; p = 0.45). As we can see, the heterogeneity rate of the analysis was significant, so we chose the random analysis model.

**Figure 5:**
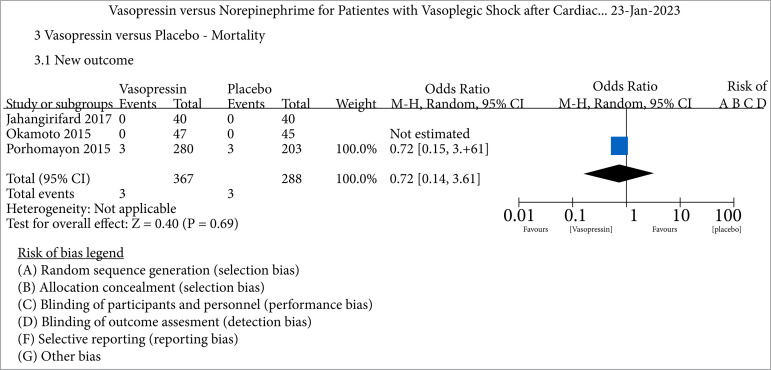
Mortality analysis comparing vasopressin versus placebo.

Source: Elaborated by the authors.

Considering the mortality outcome, we analyzed vasopressin versus placebo and found three studies, included in our analysis. As shown in the forest plot (Fig. 5), no statistically significant difference was found for the intervention (vasopressin use). However, this analysis requires attention because the event (death) did not occur in two studies, making the examination impossible.

### Length of hospital stay

**Figure 6:**
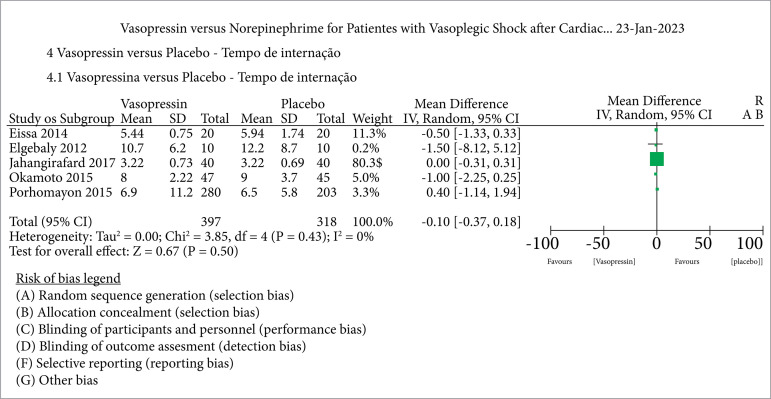
Comparison of length of stay about the use of noradrenaline and vasopressin.

Source: Elaborated by the authors.

We performed a meta-analysis using a length of stay as the endpoint. We included four studies that compared vasopressin versus norepinephrine. In this analysis, we also performed the random effect due to heterogeneity. As shown in Fig. 6, three studies had a lower mean length of stay in the vasopressin group. One study had a lower mean for the norepinephrine group, but the result of our meta-analysis showed no statistically significant difference (OR = 0.08; 95%CI 0.40–0.56, I2 = 87%; z = 0.33; p = 0.74).

**Figure 7:**
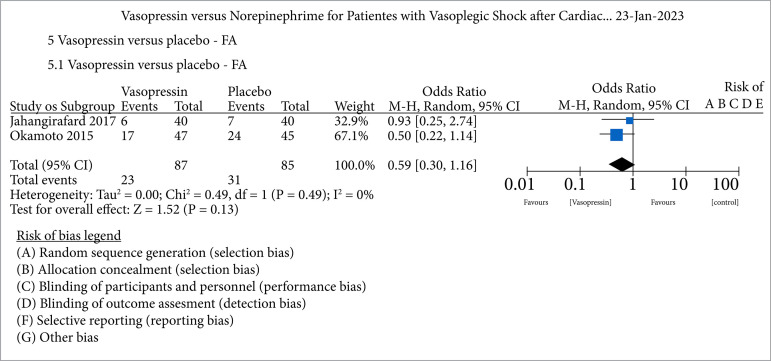
Vasopressin versus placebo concerning length of stay.

Source: Elaborated by the authors.

When analyzing vasopressin versus placebo, regarding the outcome of the length of hospital stay, five studies comprised our analysis, as shown in the forest plot (Fig. 7); there was also no statistical significance regarding the use of placebo and vasopressin (OR = -0.10; 95%CI -0.37–-0.18, I2 = 0%; z = 0.67; p = 0.50).

## Discussion

In our systematic review, we evaluated the effect of vasopressin in patients with vasoplegic shock after significant surgery. We included studies that compared vasopressin with norepinephrine and placebo and evaluated some outcomes reported in the articles as mortality and length of stay. After quantitative and qualitative synthesis of the studies, we observed that, although some studies indicate that vasopressin decreased some of the outcomes significantly, the same was not observed in others. The results are extremely heterogeneous, which can be seen in the meta-analysis performed; in none of them we observed significant differences. However, although we did not find benefits associated with the use of vasopressin, we did not find that its use brought a higher frequency of comorbidities for patients either, showing some safety for its use.

Vasopressin is a nonapeptide hormone formed by sequences of nine amino acids. This hormone is produced by magnocellular neurons located in the paraventricular and supraoptic nuclei of the hypothalamus, in which it is stored until its use^21^. This synthesis and storage cycle lasts approximately two hours. When in the bloodstream, vasopressin has a half-life of between 10 and 35 minutes and is degraded in the kidneys and liver through the action of peptidases. Compared to other vasoconstrictor agents such as noradrenaline and phenylephrine, vasopressin is considered more potent because, besides the contraction mechanism, this hormone also uses other contractile cascades to function in vascular smooth muscle. These mechanisms may contribute to the justification of its use in the clinic during vasoplegic shock. However, whether this contribution is beneficial, it is still unclear since its vasoconstrictor potential is very pronounced.

The use of vasopressin in this context has been recent yet. Argenziano et al.^22^ in 1997 aimed to evaluate through a randomized clinical trial the effect of vasopressin in the VS after implantation of a ventricular assist device in patients with advanced heart failure Ten patients were included in this clinical trial; five were randomized to vasopressin and five to saline solution. In the intervention group (vasopressin), a statistically significant improvement in mean arterial pressure and systemic vascular resistance was observed.

Forty-eight patients who developed vasodilatory shock after cardiovascular surgery were included in another previously published study. These patients were randomized into two groups; one received vasopressin, and the other group received vasopressin and noradrenaline. Hemodynamic parameters were evaluated for 48 hours, and the authors demonstrated that the group of patients treated with vasopressin had a statistically significant decrease in heart rate. In addition, no significant adverse effects were observed with vasopressin^23^.

In our systematic review, we included a single Brazilian study, a randomized clinical trial in which 82 patients were included and randomized into two groups, one group received vasopressin, and the other one norepinephrine. The outcome was assessed as the length of stay, mortality, presence of organ dysfunctions, and adverse effects. In this study, as already mentioned, a significant difference was observed in the length of stay in the ICU favorable to the intervention (vasopressin), which was approximately four days compared to seven days for noradrenaline. Also, in this study, the authors showed no significant difference considering mortality, and the group treated with vasopressin showed a lower incidence of renal failure when compared to noradrenaline^3^.

There are many studies published in the literature that had as an outcome the use of vasopressin in the clinical management of vasoplegic shock, especially after cardiac surgeries. The results of the works are controversial, which makes it crucial to perform systematic reviews especially with meta-analysis. Here, in our systematic review, we included clinical trials and also observational studies that were available and that met the previously established criteria, i.e., that included patients with VS or high risk for such a condition to define the efficacy of vasopressin in this clinical setting.

The VS in the immediate postoperative period of cardiac surgery has an incidence of 26%, and its severity is proportional to the response to vasoactive amines^24^. The main drug for treating mild to moderate syndrome is noradrenaline. Still, some evidence shows that noradrenaline alone is ineffective for a significant fraction of patients. In this sense, it is necessary to search for therapeutic alternatives, and among the drugs that are used in studies we have methylene blue and vasopressin, the drugs considered most promising^24^.

Our systematic review tried to evaluate vasopressin’s efficacy and safety in managing vasoplegic shock. We found an extremely heterogeneous scenario of clinical studies, which included six randomized clinical trials. Among these trials, three evaluated the prophylactic use of vasopressin versus placebo. The outcomes assessed in these studies were different, which makes comparison difficult. Still, in all of them, benefits were demonstrated in the use of vasopressin.

Most importantly, it was shown that its use in low doses prophylactically is safe for patients^13^^,^ 15 ^,^ 16. Only one clinical trial evaluated mortality as the primary endpoint. The results demonstrated that the use of vasopressin compared to norepinephrine was superior and decreased the death rate of patients^3^. One clinical trial evaluated the development of acute renal failure. It demonstrated no significant differences in the incidence of this condition comparing vasopressin versus noradrenaline^15^. We included four observational studies; two evaluated mortality as an endpoint comparing the use of vasopressin with noradrenaline and found no statistically significant differences^19^^,^ 20. A cohort study demonstrated that vasopressin administered in patients with mesenteric ischemia as a complication was superior to noradrenaline considering the improvement of intestinal perfusion^18^. We included a cohort study that demonstrated that using vasopressin increased the chance of developing acute renal failure by more than three times compared to matched patients who did not use the drug^17^.

We found no significant differences in any of the comparisons performed in our meta-analysis. In the literature for comparison, we found a single previously published systematic review on the subject. In this review, the authors included three studies, two randomized clinical trials and one retrospective cohort study. No differences were observed in mortality rates, ventricular arrhythmias, and duration of mechanical ventilation, however, conflicting results were observed for acute kidney injury, atrial arrhythmias, duration of vasopressors and length of ICU stay, as in our review the authors concluded that vasopressin was not a drug considered superior for any of the outcomes analyzed^26^.

Finally, it is worth noting that, although our systematic review and meta-analysis does not allow any favorable conclusion for the use of vasopressin, we could observe that only one observational study indicated that the use of vasopressin increases the risk of developing renal failure. However, this finding was not corroborated in a clinical trial conducted with the same objective.

Considering the limitations of our study, we emphasize that our results should be observed with caution, since due to the limited number of studies we included articles with different methodologies, small sample size, and in different populations. Furthermore, no subgroup analysis or adjustments were performed in the meta-analysis.

## Conclusion

We concluded that more studies, especially clinical trials, should be conducted in a methodological setting of excellence so that more robust and safer conclusions can be inferred about using vasopressin in managing vasoplegic shock.

## Data Availability

The datasets used and/or analyzed during the current study available from the corresponding author on reasonable request.
